# Development and validation of a brief form of the Anticipated Effects of Food Scale

**DOI:** 10.1016/j.appet.2024.107843

**Published:** 2024-12-24

**Authors:** Jenna R. Cummings, Natasha Treharne, Uku Vainik, Ashley E. Mason, Tonja R. Nansel, Leah M. Lipsky, Ashley N. Gearhardt

**Affiliations:** a Department of Psychology, Institute of Population Health, University of Liverpool, Liverpool, UK; b Institute of Psychology, University of Tartu, Tartu, Estonia; c Institute of Genomics, University of Tartu, Tartu, Estonia; d Montreal Neurological Institute, McGill University, Montreal, Canada; e Department of Psychiatry and Behavioral Sciences, University of California, San Francisco, San Francisco, CA, USA; f Social and Behavioral Sciences Branch, Division of Population Health Research, Eunice Kennedy Shriver National Institute of Child Health and Human Development, Bethesda, MD, USA; g Department of Psychology, University of Michigan, Ann Arbor, Ann Arbor, MI, USA

**Keywords:** Assessment, Brief, Eating behaviour, Emotions, Food-related emotional expectancies, Measurement

## Abstract

Identifying malleable influences on eating behaviours will advance our ability to improve physical and mental health. Food-related emotional expectancies are the anticipated positive and negative emotions from eating different foods and are theorised to affect eating behaviour, and to be amenable to change. The Anticipated Effects of Food Scale (AEFS) assesses food-related emotional expectancies using 62 one-word items; however, a shorter questionnaire would be useful in large and clinical studies. In the present study, we developed a brief version of the AEFS, named the AEFS-Brief (AEFS-B), using a data-driven approach. We identified candidate items from all-subset correlations with the AEFS and item-level correlations with eating behaviours in two community samples (*n* = 247, *n* = 718), and we assessed internal consistency and validity of the AEFS-B. We further assessed internal consistency and validity in two independent samples (*n* = 200, *n* = 108) that completed a ‘bogus’ taste test or 24-h dietary recalls. Results indicated that the AEFS-B with 28 one-word items had good internal consistency and convergent validity with the AEFS. Analysis with AEFS-B scores reproduced associations of AEFS scores with intake of added sugars, symptoms of food addiction, eating to cope motives, and *ad libitum* food intake. We also demonstrated novel associations of AEFS and AEFS-B scores with emotional eating and diet quality. The AEFS-B appears to be a reliable and valid brief measure of food-related emotional expectancies that can be used in cohort and population studies, ecological momentary assessments, and for clinical populations in which participant burden is high.

## 1. Introduction

Many people struggle to eat in a way that supports their health. Overeating highly processed foods, which are designed to be particularly rewarding through the addition of fat and/or refined carbohydrates (e.g., ice cream, pizza, soda; Schulte et al., 2015), and undereating unprocessed and minimally processed foods (e.g., fresh and frozen fruit and vegetables) are common behaviours. Researchers estimate more than one-third of the adult population eats in response to the negative emotion of stress rather than hunger (American Psychological Association, 2016), and 14% respond to highly processed foods like an addiction to psychoactive substances (e.g., eat more than intended, continue to eat despite adverse consequences; Praxedes et al., 2022). Collectively, these eating behaviours are associated with greater risk of metabolic syndrome, type 2 diabetes, cardiovascular disease, cancer, neurodegenerative disease, general anxiety disorder, and major depressive disorder (Burrows et al., 2018; Morze et al., 2020; Tsenkova et al., 2013). Identifying malleable influences on these eating behaviours will advance our ability to improve physical and mental health.

Expectancy theory proposes that through social and personal learning individuals store in memory the outcomes of a behaviour, developing “expectancies” that subsequently influence their future behaviour (Bandura & Walters, 1977; Goldman et al., 1999; James et al., 1890). For example, after feeling very joyful when drinking alcohol with family, individuals may develop a strong expectancy that they would feel joy each time they drink alcohol, and as a result, drink more frequently and heavily in the future. Over 100 studies demonstrate stronger positive alcohol-, cigarette-, and sedentary-related emotional expectancies are linked with more alcohol use, cigarette smoking, and sedentary behaviour, respectively (Monk & Heim, 2013; Patel & Fromme, 2010; Williams et al., 2005). Decades of research findings also indicate strong positive alcohol-related emotional expectancies are overestimated, and modifiable via expectancy challenge (Darkes & Goldman, 1993; Gesualdo & Pinquart, 2021). Applying expectancy theory to eating behaviours may be beneficial for science and practice.

Food-related emotional expectancies are the anticipated positive and negative emotions from eating different foods (Cummings et al., 2020). In accordance with expectancy theory, stronger positive highly-processed-food expectancies (e.g., “I expect to feel happy while eating pizza”) were moderately to strongly associated with greater self-reported intake of added sugars, symptoms of food addiction, and eating to cope motives, and greater intake of highly relative to minimally processed food observed in a simulated fast-food restaurant (Cummings et al., 2020, 2021, 2023). Also, brief experimental manipulations, such as exposure to a minute-long collection of video food advertisements, have affected the strength of positive highly-processed-food expectancies (Cummings et al., 2021, 2023). These initial findings suggest that positive highly-processed-food expectancies are malleable influences on eating behaviours.

The Anticipated Effects of Food Scale (AEFS), a self-report questionnaire, is the existing assessment of food-related emotional expectancies for adults and has demonstrated good internal consistency and convergent, discriminant, incremental, and criterion validity (Cummings et al., 2020). Respondents rate the extent to which they expect to feel 15 positive emotions while eating highly processed foods. They also rate the extent to which they expect to feel 16 negative emotions while eating highly processed food, though negative substance-related emotional expectancies are inconsistently linked with less substance use (Monk & Heim, 2013; Patel & Fromme, 2010), and sometimes linked with greater substance use (Mann et al., 1987; McMahon et al., 1994). In addition, respondents rate the extent to which they expect to feel the same 31 emotions while eating unprocessed/minimally processed foods. The measure addresses both highly processed foods and unprocessed/minimally processed foods because their intakes have additive effects on health (Kennedy et al., 1995), and assessing emotional expectancies for both food groups allows for exploration of their joint role in influencing eating behaviours.

The AEFS comprises 62 one-word items, assessing a broad range of positive and negative emotional expectancies for both highly processed foods and unprocessed/minimally processed foods (Cummings et al., 2020). A version with fewer items would hold several advantages for use in studies with limited space or time, including population and cohort studies with large batteries of questionnaires and ecological momentary assessments with repeated sampling at multiple time points. A shorter version could also be preferable for clinical populations in which participant burden is high (e.g., hospital patients). The ideal brief version would capture all four groups of food-related emotional expectancies precisely while recreating the normal distribution of the original version (Smith et al., 2016).

The aim of the present study was to develop a brief version of the AEFS, named the AEFS-Brief (AEFS-B), using a data-driven approach. We tested its internal consistency, and we evaluated its validity by examining associations of the AEFS-B with the AEFS and with eating behaviours. We accomplished this aim using data collected online and in-person from different populations across multiple projects, maximising generalisability of the findings.

## 2. Methods

### 2.1. Study design

We designed the present study based on the methods used to develop a brief version of the Reward-based Eating Drive questionnaire (Vainik et al., 2019). We conducted a secondary analysis of data from four projects (Cummings et al., 2020, 2021, 2023; Nansel et al., 2016). Each project included assessment of (1) food-related emotional expectancies via the AEFS and (2) at least one eating behaviour. To reproduce associations of the AEFS using the AEFS-B, eating behaviours included those previously examined in relation to AEFS scores (i.e., intake of added sugars, symptoms of food addiction, eating to cope motives, and *ad libitum* food intake; Cummings et al., 2023; Cummings et al., 2021; Cummings et al., 2020). To extend previous research, eating behaviours also included those that had not been studied in relation to AEFS scores (i.e., emotional eating and diet quality).

### 2.2. Participants

Please see the original projects for information on data quality check procedures (e.g., excluding from analysis participants who incorrectly answered quality control questions in online studies) and number of participants excluded at each stage before analysis (Cummings et al., 2020, 2021, 2023; Nansel et al., 2016). Table 1 summarises sample demographics for analytic samples from Projects 1–4. Projects 1 and 2 involved community samples recruited via Amazon’s Mechanical Turk platform on February 13th, 2019, and August 11th–12th, 2020, respectively (Cummings et al., 2020, 2023). Project 3 included Midwestern university students recruited through the Department of Psychology’s subject pool from January to December 2019 (Cummings et al., 2021). Project 4 involved birthing parents from North Carolina participating in a follow-up visit of the Pregnancy Eating Attributes Study from October 2021 to May 2023, when their children were about 6 years old (Nansel et al., 2016).

### 2.3. Procedures

All procedures were approved by respective institutional review boards in accordance with the provisions of the World Medical Association Declaration of Helsinki (see Cummings et al., 2023; Cummings et al., 2021; Cummings et al., 2020; Nansel et al., 2016 for full procedure details). In Project 1, participants provided informed consent and completed the AEFS, the National Cancer Institute’s Dietary Screener Questionnaire, the modified Yale Food Addiction Scale 2.0, the Dutch Eating Behaviour Questionnaire and the Palatable Eating Motives Questionnaire. In Project 2, participants were randomly assigned to watch video advertisements for food or cell phones and all participants completed the AEFS and the modified Yale Food Addiction Scale 2.0. In Project 3, participants were randomly assigned to sit in a simulated fast-food restaurant or an office, and all participants completed the AEFS, the Palatable Eating Motives Scale, and a ‘bogus’ taste test wherein *ad libitum* food intake was measured. In Project 4, participants completed the AEFS and a 24-h dietary recall at a scheduled study visit, and another 24-h dietary recall was conducted approximately ten days later; we used 24-h dietary recall data to determine diet quality.

### 2.4. Measures

#### 2.4.1. Anticipated Effects of Food Scale (AEFS)

The AEFS is a 62-item questionnaire assessing food-related emotional expectancies (Cummings et al., 2020). Respondents are asked to imagine eating highly processed foods and unprocessed/minimally processed foods and rate how much they expect to feel 15 positive and 16 negative emotions while eating these foods. Respondents rate the items on a 6-point Likert Scale from 1 (*Definitely not*) to 6 (*Definitely*). In Projects 1–3, we labelled food groups with the colloquial terms ‘junk’ and ‘healthy’ and provided example foods [i.e., “Imagine that you are eating junk food (e.g., sweets, salty snacks, fast foods, sugary drinks) …” and “Imagine that you are eating healthy food (e.g., fruits, vegetables) …“]. In Project 4, we removed the labels to reduce potential social desirability bias (e.g., “Imagine that you are eating foods like sweets, salty snacks, fast foods, or sugary drinks …” and “Imagine that you are eating foods like fruits or vegetables …“).

#### 2.4.2. National Cancer Institute’s Dietary Screener Questionnaire

The National Cancer Institute’s Dietary Screener Questionnaire is a 26-item questionnaire designed to quickly estimate intake of certain nutrients over the past month (Thompson et al., 2017). A subset of 9 items assesses added sugars intake via participants reporting the intake frequency of soda, fruit drinks, cookies/cakes/pies, doughnuts, ice cream, sugar/honey in coffee/tea, candy, and certain cereals. We estimated daily added sugars intake by applying publicly available scoring algorithms that couple the frequency responses with sex- and age-specific portion size information (Thompson et al., 2017).

#### 2.4.3. Modified Yale Food Addiction Scale 2.0

The 13-item modified version of the Yale Food Addiction Scale 2.0 measures addictive-like responses to highly processed food aligned with the Diagnostic and Statistical Manual of Mental Disorders (5th ed.) criteria for substance use disorders (Schulte & Gearhardt, 2017). Respondents consider their difficulty in controlling intake of these foods over the past year. Sample items are, “Eating the same amount of food did not give me as much enjoyment as it used to,” and, “I avoided work, school or social activities because I was afraid I would overeat there.” Respondents answer on an 8-point Likert scale from 1 (*Never*) to 8 (*Every day*). We used established scoring practices to determine if a respondent met the “diagnostic” threshold for a symptom of substance use disorder (with relevance to highly processed food). We summed symptom scores to generate a dimensional score representing severity of food addiction (Schulte & Gearhardt, 2017).

#### 2.4.4. Dutch Eating Behaviour Questionnaire

The Dutch Eating Behaviour Questionnaire is a 33-item questionnaire that assesses different patterns of eating behaviour (Van Strien et al., 1986). The Emotional Eating subscale includes 13 items assessing the frequency that a person desires to eat in response to their negative emotions. Sample items are “Do you have the desire to eat when you are irritated?” and “Do you have a desire to eat when you are bored or restless?” Respondents answer items on a 5-point Likert scale from 1 (*Never*) to 5 (*Very Often*).

#### 2.4.5. Palatable Eating Motives Scale

The Palatable Eating Motives Scale is a 20-item questionnaire that assesses different reasons for eating highly processed foods (Burgess et al., 2014). The Coping subscale includes 4 items assessing the extent to which a person eats these foods to cope with their negative emotions (e.g., “to forget your worries”). Respondents answer items on a 5-point Likert scale from 1 (*Never*) to 5 (*Always*).

#### 2.4.6. Ad libitum food intake

In the ‘bogus’ taste test, participants are instructed to taste and rate foods without awareness that their food intake is measured (Robinson et al., 2017). In Project 3, participants tasted one Oreo mini cookie (from ~25), one Lay’s plain potato chip (from ~20), one baby carrot (from ~20), and one grape (from ~25) in their preferred order, and then were left alone for 5 min to help themselves to the remaining food (see Cummings et al., 2021 for nutrition information). Research assistants weighed the food in grams before and after the test and subtracted the post-weight of each food item from the pre-weight to determine total grams consumed. We multiplied total grams consumed by the calorie content per gram specific to each food item, and we divided energy intake of the highly processed food (i.e., cookies + potato chips) by energy intake of the minimally processed food (i.e., grapes + carrots). Higher values indicated greater energy intake from highly relative to minimally processed foods.

#### 2.4.7. Diet quality

Registered dietitians administered two 24-h dietary recalls using the Nutrition Data System for Research software developed by the Nutrition Coordinating Center, University of Minnesota, Minneapolis, MN (Sievert & Buzzard, 1988). The 24-h dietary recall method is considered the least biased self-report measure of dietary intake available (Prentice et al., 2011). Participants reported to the interviewers all foods and beverages consumed from midnight to midnight the previous day, including preparation method, food source, and portion size. Immediately after the recall, interviewers resolved any errors or unknown foods by asking follow-up questions or selecting defaults provided in Nutrition Data System for Research where appropriate. Additionally, after completion of all recalls across participants, 25% of records as well as records with data 1 standard deviation (SD) above or below the mean daily energy for participants were reviewed for potential errors.

Data from multiple recalls were summed across all days per participant. The simple scoring algorithm calculated the Healthy Eating Index-2015 (HEI-2015), an assessment of adherence to the 2015–2020 Dietary Guidelines for Americans (Krebs-Smith et al., 2018). The HEI-2015 score was calculated, summing across 9 “adequacy” components (total fruits, whole fruits, total vegetables, greens and beans, whole grains, dairy, total protein foods, seafood and plant proteins, fatty acids) and 4 “moderation” components (refined grains, sodium, added sugars, saturated fats), with a range from 0 to 100. Higher values indicate higher diet quality through closer adherence to dietary guidelines.

#### 2.4.8. Demographics

Participants self-reported their age (in years), height, and weight and selected their sex assigned at birth (or gender, depending on the question wording in the project), highest education level, and their race/ethnicity from multiple categories. In Project 3, university students reported their parent’s highest education level. In Project 4, female sex assigned at birth was assumed due to pregnancy, height and weight were measured and demographics were reported during early pregnancy, with age and weight measurements updated at each subsequent visit (Nansel et al., 2016). Across studies, we dummy-coded race/ethnicity (0 = white and non-Hispanic and 1 = non-white and/or Hispanic) and computed body mass index (kg/m^2^).

### 2.5. Data analysis

Data for Projects 1–3 are available via the Open Science Framework: https://osf.io/mdq9p/. Data for Project 4 are available upon request. We conducted analysis in R version 4.4.1 (Vienna, Austria) utilising the psych, tidyverse and cowplot packages and SPSS version 29.0.1.0, with syntax available on the Open Science Framework site.

To inform the development of the AEFS-B, we first conducted all-subset correlation analysis using data from Projects 1 and 2 and averaged correlation values to obtain a more stable estimate. All-subset correlation analysis identifies, from all possible combinations of items, the shortest scale that maximally discriminates between respondents in a normal distribution (Smith et al., 2016). We plotted the potential numbers of items of the AEFS-B (x-axis) against correlations with the AEFS (y-axis) and identified the “elbow” in the emerging brevity-correlation trade-off. We conducted analysis separately for items from each subscale since there is no overall AEFS score (Cummings et al., 2020). To identify top-performing items, we ranked subsets based on the strength of their correlations with the AEFS and noted the items that most frequently appeared in the subsets that had the strongest average correlations across all four subscales.

In addition to all-subset correlation analysis, we next conducted bivariate correlations between individual items of the AEFS and eating behaviours using data from Projects 1 and 2 to identify other items that could improve the performance of the AEFS-B and widen the coverage of affective space. The circumplex model of affect posits that emotions vary in two factors: (1) valence, which is the extent to which emotions include pleasurable or unpleasurable experiences and (2) arousal, which is the extent to which emotions include activated or deactivated experiences (Posner et al., 2005). The variety of interactions between valence and arousal among emotions are mapped onto a circular graph divided into four quadrants representing interactions. For example, “happy” would be mapped as a “high-arousal highly positive” emotion, “calm” would be mapped as a “low-arousal positive” emotion, “worried” would be mapped as a “high-arousal negative” emotion, and “depressed” would be mapped as a “low-arousal highly negative” emotion. Since we developed the original AEFS to have representation of diverse emotions according to this model (Cummings et al., 2020), we sought this representation for the AEFS-B too.

After selecting candidate items for the AEFS-B, we computed “positive highly-processed-food expectancy,” _“_negative highly-processed-food expectancy,” _“_positive minimally-processed-food expectancy,” and “negative minimally-processed-food expectancy” scores in data from Projects 1–4 by taking the average across the respective items; we evaluated internal consistencies by estimating Cronbach’s alpha and assessed convergent validity by examining the strength of bivariate correlations with AEFS scores. We then evaluated criterion validity by conducting bivariate correlations of the AEFS-B scores with intake of added sugars, symptoms of food addiction, emotional eating, eating to cope motives, *ad libitum* food intake, and diet quality, comparing the strength, direction, and statistical significance of the correlations with those of the correlations between AEFS scores and eating behaviours. Due to the demographic heterogeneity across samples, we residualized eating behaviour variables for age, highest level of education (for self or parent) and race/ethnicity. We residualized eating behaviour variables for sex assigned at birth (or gender) in Projects 1–3; in Project 4, the entire sample was female. We used complete case analysis because of little missing data (<3% in each dataset). We set the statistical significance threshold at *p* < .05.

## 3. Results

### 3.1. Candidate items for AEFS-B

**Fig. 1.**
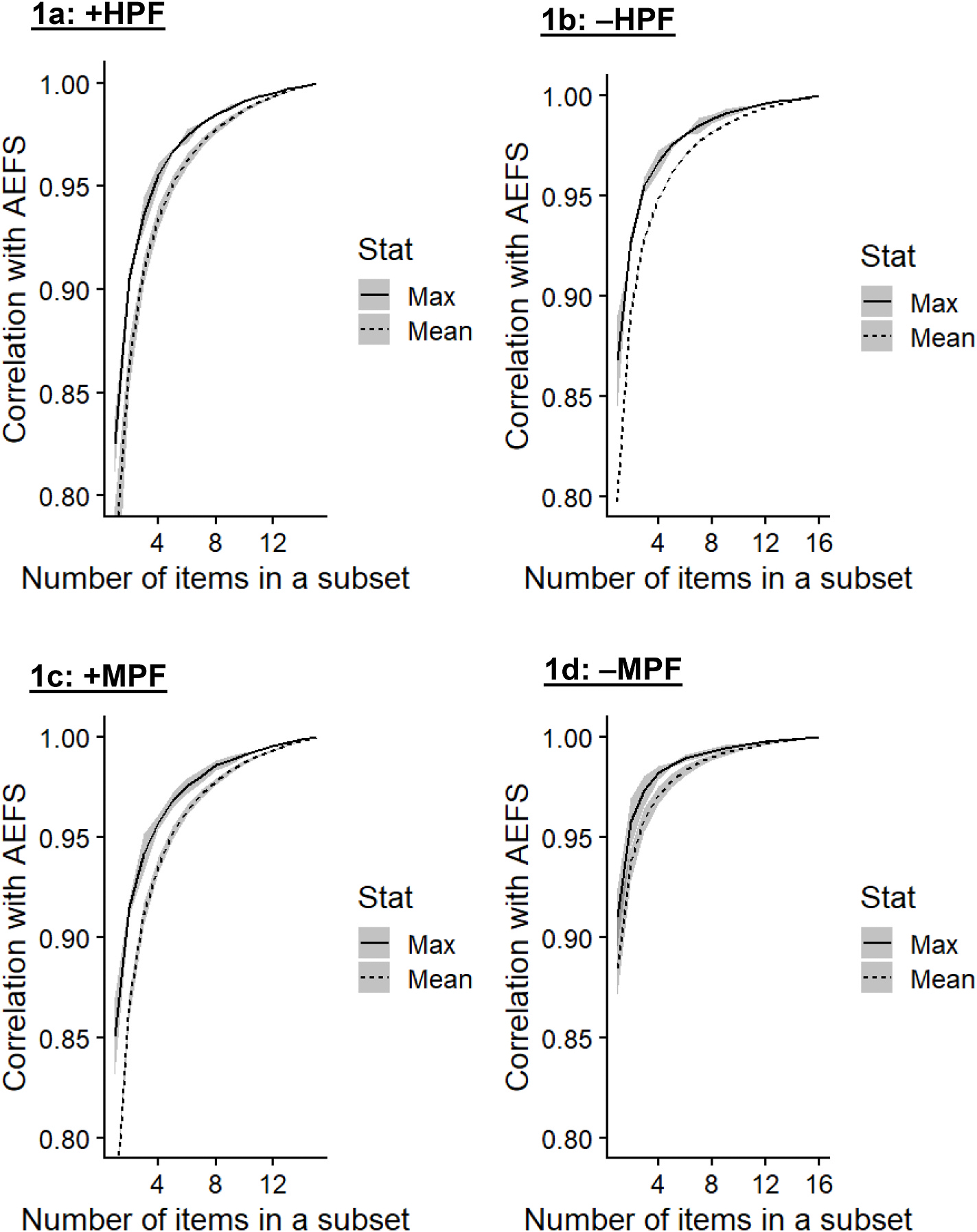
a–d. Mean and maximum correlations between scores derived from item subsets and AEFS scores. Lines represent estimates when correlations are averaged between data from Projects 1 and 2. Borders of grey ribbons denote individual values in Projects 1 or 2 (variability between samples). AEFS = Anticipated Effects of Food Scale, +HPF = Positive highly-processed-food expectancy score, –HPF = Negative highly-processed-food expectancy score, +MPF = Positive minimally-processed-food expectancy score, –MPF = Negative minimally-processed-food expectancy score.

Table S1 in Supplemental Materials presents means and standard deviations of the AEFS items. In the all-subset correlation analysis using data from Projects 1 and 2, we generated 32,767 possible item subsets for each positive expectancy subscale and 65,535 for each negative expectancy subscale. Fig. 1a–d displays mean and maximum correlations between scores derived from the subsets and AEFS scores. Elbows in potential numbers of items plotted against correlations with the AEFS scores ranged from 4 to 7. For consistency across subscales, and to maximize reliability, we opted for 7 items for each subscale in the AEFS-B, which would total to 28 items. The AEFS-B would thus include less than half of the items from the original version. Top-performing items from the all-subset correlation analysis across all subscales included “relieved,” “cheerful,” “comforted,” “refreshed,” “relaxed,” “frustrated,” “down,” “tired,” “depressed,” and “numb.”

Tables S2–3 in Supplementary Materials present bivariate correlations between individual items of the AEFS and eating behaviours using data from Projects 1 and 2. Results indicated that the items that performed well in the all-subset correlation analysis showed moderate to strong associations with eating behaviours. The lead and senior author next, with reference to the circumplex model of affect (Posner et al., 2005), noted how the top-performing items from all-subset correlation analysis were mostly low-arousal positive and negative emotions, and among the negative emotions, there was little variety in degree of valence. Items that would widen the coverage of affective space including “happy” (adding high-arousal positive), “excited” (adding high-arousal positive), “anxious” (adding high-arousal negative) and “bored” (adding more neutral negativity) showed some of the strongest associations with eating behaviours for the highly- or minimally-processed-food subscales. We therefore added these items with the top-performing items from the all-subset correlation analysis to create the AEFS-B.

### 3.2. Internal consistency and validity

Table 2 presents the means and standard deviations, Cronbach’s alpha estimates, and bivariate correlations of the AEFS-B scores with the AEFS scores for each subscale using data from Projects 1–4. All Cronbach’s alphas were >.75, and all correlations of the AEFS-B scores with respective AEFS scores were >.92, indicating acceptable internal consistency and convergent validity.

Table 3presents bivariate correlations of the AEFS and AEFS-B scores with eating behaviours using data from Projects 1–4. Correlations of the AEFS-B scores with eating behaviours had the same direction, magnitude, and statistical significance as correlations of the AEFS scores with eating behaviours. Greater positive highly-processed food expectancies, whether assessed by the AEFS or AEFS-B, were associated with greater intake of added sugars (small effect sizes), greater symptoms of food addiction (large effect sizes), greater emotional eating (large effect sizes), greater eating to cope motives (medium to large effect sizes), greater energy intake from highly relative to minimally processed food (medium effect sizes), and worse diet quality (small effect sizes).

Greater negative highly-processed-food expectancies, whether assessed by the AEFS or AEFS-B, were associated with greater intake of added sugars (small effect sizes), greater symptoms of food addiction (large effect sizes), greater emotional eating (large effect sizes), and greater eating to cope motives (medium effect sizes); correlations with *ad libitum* food intake and diet quality were non-significant. With regards to minimally processed food expectancies assessed by the AEFS or AEFS-B, greater positive expectancies were associated with greater symptoms of food addiction (small effect sizes) and greater emotional eating (small effect sizes) but with less energy intake from highly relative to minimally processed food (small effect sizes); correlations with intake of added sugars, eating to cope motives, and diet quality were non-significant or inconsistently significant across studies. Greater negative minimally-processed-food expectancies were associated with greater intake of added sugars (small effect sizes), greater symptoms of food addiction (large effect sizes), greater emotional eating (large effect sizes), greater eating to cope motives (medium to large effect sizes), and greater energy intake from highly relative to minimally processed food (small effect sizes); correlations with diet quality were non-significant.

## 4. Discussion

We developed a brief measure of food-related emotional expectancies by evaluating the trade-off in brevity versus correlation with the original measure for different item combinations. The resulting AEFS-B includes 28 one-word items, less than half of the AEFS, and demonstrated good internal consistency and convergent and criterion validity. This shorter measure holds promise for rapid assessment of food-related emotional expectancies in studies with limited space or time, and for clinical populations in which participant burden is high. The AEFS-B, including instructions for its scoring, are available on the Open Science Framework: https://osf.io/mdq9p/.

Associations of the AEFS-B with eating behaviours were like those observed with the AEFS. Stronger positive highly-processed-food expectancies were moderately to strongly associated with greater self-reported intake of added sugars, symptoms of food addiction, and eating to cope motives, and greater observed intake of highly relative to minimally processed food (Cummings et al., 2020, 2021, 2023). Extending prior work, stronger positive highly-processed-food expectancies (assessed by the AEFS or the AEFS-B) were also moderately associated with worse diet quality assessed by 24-h dietary recall, which is considered the least biased self-report measure of dietary intake available (Prentice et al., 2011), and were strongly associated with greater emotional eating. It is noteworthy that the tendency to eat in response to *negative* emotions was linked with stronger positive, rather than weaker negative, highly-processed-food expectancies, and future research on the role of food-related emotional expectancies in emotional eating is warranted given that more than one-third of the adult population engages in this behaviour with regards to their stress (American Psychological Association, 2016). Associations of positive highly-processed-food expectancies with multiple self-reported and observed eating behaviours in different samples strongly supports application of expectancy theory in the domain of eating behaviour.

Negative highly-processed-food expectancies and positive and negative minimally-processed-food expectancies measured by the AEFS-B also showed small to large associations with some eating behaviours. Expectancy theory does not definitively address how valence of emotional expectancies is implicated in behaviours, though researchers proposed associations between negative substance expectancies and substance use vary as a function of substance use disorder severity (Mann et al., 1987; McMahon et al., 1994). For individuals who have experienced severe negative consequences of substance use, both stronger positive and negative substance expectancies are linked with more substance use (Mann et al., 1987). Results that both stronger positive and negative highly-processed-food expectancies were largely associated with more symptoms of food addiction are consistent with this logic. Expectancy theory also does not address the joint role of different substance expectancies in influencing behaviour, likely because there are no “unprocessed/minimally processed” alcohols/cigarettes/etc. Findings that positive and negative minimally-processed-food expectancies were moderately associated, respectively, with less and greater intake of highly relative to minimally processed foods suggest they could play an important role in overeating highly processed foods too. Including assessment of all four expectancy groups in both the AEFS and the AEFS-B allows researchers to investigate their independent and interactive influences on eating behaviours.

The large associations of food addiction and emotional eating scores with some types of food-related emotional expectancies might indicate measures are capturing the same latent construct. Although food-related emotional expectancies are related to eating behaviours, the AEFS/AEFS-B requires individuals to think about the anticipated consequences of their eating behaviour (e.g., “I expect to feel down while eating fruit and vegetables”), whereas food addiction instruments require individuals to think about frequency of behaviours and distress (e.g., “I had significant problems in my life because of food and eating”) and emotional eating measures require individuals to think about frequency of behaviours (e.g. “Do you have the desire to eat when you are irritated?“) (Cummings et al., 2020; Schulte & Gearhardt, 2017; Van Strien et al., 1986). Furthermore, emotional eating measures assess how emotions that occur *before* eating impact the behaviour. Although food addiction and emotional eating measures identify individuals with greater risk of metabolic syndrome, type 2 diabetes, general anxiety disorder, and major depressive disorder (Burrows et al., 2018; Tsenkova et al., 2013), they do not identify the cognitive-affective mechanisms by which eating behaviours are reinforced. By identifying the specific food-related emotional expectancies implicated in clinically relevant eating behaviours, we can target changing those expectancies with novel cognitive-affective techniques. For example, like done with alcohol-related expectancy challenge, individuals could rate their positive food-related emotional expectancies before eating, rate their positive emotions while eating, and receive feedback on over-estimations. Meta-analysis across 23 studies (N = 4122) indicates that receiving this kind of feedback with regards to alcohol use robustly has reduced positive alcohol-related emotional expectancies, thereby reducing heavy alcohol use in adolescents and adults (Gesualdo & Pinquart, 2021).

Results should be interpreted considering study strengths and limitations. We applied the sophisticated all-subset correlation method in two large datasets to inform AEFS-B item selection. We avoided typical limitations of scale-shortening methods by preserving the AEFS-B expectancy/affective coverage of the original measure, assessing its internal consistency and overlap with the AEFS, and evaluating its validity against multiple eating behaviours in four datasets, including two independent from those that informed item selection (Smith et al., 2000). However, the latter datasets included data from students and birthing parents, so further validation in more diverse populations would expand the generalisability of the study results. Test-retest reliability has not been tested for the AEFS and AEFS-B, and different versions will be needed to assess food-related emotional expectancies in children. The lack of longitudinal investigations of food-related emotional expectancies is a key gap because expectancy theory proposes expectancies begin forming in childhood and become relatively stable in adulthood (Smith & Goldman, 1994). The AEFS and AEFS-B assess food-related emotional expectancies for two broad food categories. Descriptions of food categories consistent with approaches like the quantitative definition for “hyper-palatable foods” (Fazzino et al., 2019) or the NOVA classification system definition of “ultra-processed foods” (Monteiro et al., 2019) could promote synergy of research on food-related emotional expectancies with nutrition science. Future research could test the incremental utility of scores from AEFS/AEFS-B versions with different descriptions of food categories, compared to scores from the original, in predicting eating behaviours.

In conclusion, the AEFS-B appears to be a reliable and valid brief measure of food-related emotional expectancies. When space or time is flexible, or where investigating food-related emotional expectancies is the primary aim of the study, we recommend using the original version of the AEFS so a broader range of emotional expectancies can be examined. We intend for the AEFS-B to be used in ecological momentary assessments, in large population and cohort studies, and for clinical populations in which participant burden is high. Research in this area applying those study designs will yield new knowledge on the role of food-related emotional expectancies in mental and physical health.

## Supplementary Material

Supplementary Table 1

Supplementary Tables 2 & 3

Appendix A. Supplementary data

Supplementary data to this article can be found online at https://doi.org/10.1016/j.appet.2024.107843.

## Figures and Tables

**Table 1:** Demographics of analytic samples for Projects 1–4.

	Project 1 (*n* = 247)	Project 2 (*n* = 718)	Project 3 (*n* = 200)	Project 4 (*n* = 108)
	*M(SD) or n (%)*	*M(SD) or n (%)*	*M(SD) or n (%)*	*M(SD) or n (%)*

Age (years)	36.84 (11.27)	35.88 (11.40)	18.78 (1.13)	38.07 (4.06)
Sex assigned at birth^a^				
Female	115 (46.7%)	295 (41.1%)	127 (63.5%)	108 (100.0%)
Male	131 (53.3%)	416 (57.9%)	73 (36.5%)	0 (0.0%)
Non-binary	–	2 (0.3%)	–	–
Prefer not to answer	–	5 (0.7%)	–	–
Highest education level^b^				
Less than high school	0 (0.0%)	1 (0.1%)	5 (2.5%)	2 (2.1%)
High school graduate	30 (12.1%)	37 (5.2%)	14 (7.0%)	4 (4.2%)
Some college	45 (18.2%)	85 (11.8%)	19 (9.5%)	9 (9.5%)
Associate’s degree	24 (9.7%)	31 (4.3%)	4 (2.0%)	32 (33.7%)
Bachelor’s degree	117 (47.4%)	419 (58.4%)	52 (26.0%)	30 (31.6%)
Advanced degree	31 (12.6%)	139 (19.4%)	106 (53.0%)	18 (18.9%)
Prefer not to answer	–	6 (0.8%)	–	–
Race/ethnicity^c^				
Non-white and/or Hispanic	63 (25.5%)	294 (41.0%)	76 (38.0%)	20 (20.4%)
White and non-Hispanic	184 (74.5%)	401 (55.8%)	124 (62.0%)	78 (79.6%)
Prefer not to answer	–	23 (3.2%)	–	–
Body mass index (kg/m^2^)	26.28 (5.86)	24.72 (6.91)	23.21 (3.76)	27.32 (6.40)

Notes.

a In Project 2, adults self-reported their gender rather than sex assigned at birth because of the question wording and had the option to select “prefer not to answer” and, in Project 4, female sex assigned at birth was assumed due to pregnancy.

b In Project 2, adults had the option to select “prefer not to answer” and, in Project 3, university students reported their parent’s highest education level.

c In Project 2, adults had the option to select “prefer not to answer.”

**Table 2:** Means and standard deviations, Cronbach’s alphas, and correlation coefficients of AEFS-B scores with AEFS scores.

	*M*(*SD*)	Cronbach’s alpha	Correlations with:			
			AEFS +HPF	AEFS −HPF	AEFS +MPF	AEFS −MPF

**Project 1**						
	
AEFS-B +HPF	3.38 (1.25)	.90	**.98** ***	35***	.42***	.64***
AEFS-B −HPF	2.69 (1.40)	.93		**.98** ***	.42***	.75***
AEFS-B +MPF	3.87 (1.25)	.91			**97** ***	27***
AEFS-B −MPF	2.19 (1.45)	.96				**.99** ***
	
**Project 2**						
	
AEFS-B +HPF	4.20 (1.17)	.90	**.96** ***	.50***	.61***	.62***
AEFS-B −HPF	3.38 (1.38)	.91		**.98** ***	.40***	.82***
AEFS-B +MPF	4.36 (1.13)	.92			**96** ***	.38***
AEFS-B −MPF	3.08 (1.49)	.94				**.99** ***
	
**Project 3**						
	
AEFS-B +HPF	3.11 (0.71)	.79	**.96** ***	−.23**	.02	.16
AEFS-B −HPF	2.54 (0.84)	.85		**94** ***	.32***	.38***
AEFS-B +MPF	3.60 (0.78)	.85			**.95** ***	−.19**
AEFS-B −MPF	1.87 (0.52)	.76				**.94** ***
	
**Project 4**						
	
AEFS-B +HPF	2.81 (0.77)	.84	**97** ***	−.06	.39***	.13
AEFS-B −HPF	2.18 (0.77)	.84		**93** ***	.14	.56***
AEFS-B +MPF	3.19 (0.97)	.89			**.96** ***	−.03
AEFS-B −MPF	1.64 (0.66)	.91				**.98** ***

*Notes:* AEFS-B = Anticipated Effects of Food Scale-Brief, AEFS = Anticipated Effects of Food Scale, +HPF = Positive highly-processed-food expectancy score, −HPF = Negative highly-processed-food expectancy score, +MPF = Positive minimally-processed-food expectancy score, −MPF = Negative minimally-processed-food expectancy score

* *p* < .05

** *p* < .01

*** *p* < .001.

**Table 3:** Correlation coefficients of AEFS-B and AEFS scores with eating behaviours.

	Intake of added sugars	Symptoms of food addiction	Emotional eating	Eating to cope motives	*Ad libitum* food intake	Diet quality

**Project 1**						
	
+HPF: AEFS	.15*	.50***	.48***	.51***	–	–
AEFS-B	.16*	.48***	.49***	.52***	–	–
−HPF: AEFS	.17**	.56***	.53***	.45***	–	–
AEFS-B	.20**	.57***	.55***	.48***	–	–
+MPF: AEFS	−.03	.24***	.19**	.16*	–	–
AEFS-B	−.01	.24***	.19**	.16**	–	–
−MPF: AEFS	24***	.73***	.54***	.56***	–	–
AEFS-B	24***	.71***	.56***	.57***	–	–
	
**Project 2**						
	
+HPF: AEFS	–	.53***	–	–	–	–
AEFS-B	–	.47***	–	–	–	–
−HPF: AEFS	–	.58***	–	–	–	–
AEFS-B	–	.60***	–	–	–	–
+MPF: AEFS	–	.33***	–	–	–	–
AEFS-B	–	.33***	–	–	–	–
−MPF: AEFS	–	.71***	–	–	–	–
AEFS-B	–	.69***	–	–	–	–
	
**Project 3**						
	
+HPF: AEFS	–	–	–	.25***	.28***	–
AEFS-B	–	–	–	.30***	.29***	–
−HPF: AEFS	–	–	–	.21**	−.05	–
AEFS-B	–	–	–	.24***	−.06	–
+MPF: AEFS	–	–	–	.04	−.16*	–
AEFS-B	–	–	–	.06	−.16*	–
−MPF: AEFS	–	–	–	.30***	.24***	–
AEFS-B	–	–	–	.29***	.23**	–
	
**Project 4**						
	
+HPF: AEFS	–	–	–	–	–	−.21*
AEFS-B	–	–	–	–	–	−.24*
−HPF: AEFS	–	–	–	–	–	−.02
AEFS-B	–	–	–	–	–	−.05
+MPF: AEFS	–	–	–	–	–	.17
AEFS-B	–	–	–	–	–	.14
−MPF: AEFS	–	–	–	–	–	−.15
AEFS-B	–	–	–	–	–	−.18

*Notes:* Eating behaviour variables were residualised for age, highest level of education (for self or parent) and race/ethnicity. Eating behaviour variables were residualised for sex assigned at birth (or gender) in Projects 1–3; in Project 4, the entire sample was assumed female due to pregnancy. AEFS-B = Anticipated Effects of Food Scale-Brief, AEFS = Anticipated Effects of Food Scale, +HPF = Positive highly-processed-food expectancy score, −HPF = Negative highly-processed-food expectancy score, +MPF = Positive minimally-processed-food expectancy score, −MPF = Negative minimally-processed-food expectancy score.

* *p* < .05

** *p* < .01

*** *p* < .001.

## Data Availability

Data for Projects 1–3 are available via the Open Science Framework: https://osf.io/mdq9p/. Data for Project 4 are available upon request.
