# Supplementary material for: Development and validation of a brief form of the Anticipated Effects of Food Scale
Source: Appetite. Author manuscript; Available in PMC 2026 Feb 1. (PMC12314748; doi:10.1016/j.appet.2024.107843)
Supplement: Supplementary Table 1 [file NIHMS2098013-supplement-Supplementary_Table_1.docx]

Table S1

*Means and standard deviations of Anticipated Effects of Food Scale items*

|  | **Project 1** | | **Project 2** | | **Project 3** | | **Project 4** | |
| --- | --- | --- | --- | --- | --- | --- | --- | --- |
|  | **HPF** | **MPF** | **HPF** | **MPF** | **HPF** | **MPF** | **HPF** | **MPF** |
|  | *M(SD)* | *M(SD)* | *M(SD)* | *M(SD)* | *M(SD)* | *M(SD)* | *M(SD)* | *M(SD)* |
| **Positive** |  |  |  |  |  |  |  |  |
| Alert | 3.25(1.62) | 3.78(1.63) | 3.70(1.57) | 3.88(1.52) | 2.32(0.91) | 3.23(1.21) | 2.26(1.06) | 2.86(1.37) |
| Calm | 3.72(1.41) | 4.15(1.38) | 4.12(1.37) | 4.27(1.37) | 3.25(1.06) | 3.57(0.94) | 2.64(1.09) | 3.31(1.21) |
| **Cheerful** | **3.40(1.52)** | **4.06(1.46)** | **4.26(1.43)** | **4.48(1.36)** | **3.21(0.97)** | **3.66(1.05)** | **3.10(1.07)** | **3.37(1.16)** |
| **Comforted** | **3.73(1.50)** | **3.63(1.54)** | **4.39(1.32)** | **4.14(1.47)** | **3.47(1.10)** | **3.13(1.09)** | **3.19(1.14)** | **3.00(1.26)** |
| Content | 3.79(1.47) | 4.17(1.47) | 4.29(1.30) | 4.23(1.40) | 3.98(1.14) | 4.12(1.12) | 3.48(1.17) | 3.80(1.19) |
| Energized | 2.99(1.58) | 4.16(1.43) | 3.83(1.65) | 4.68(1.26) | 2.57(1.05) | 4.18(1.07) | 2.56(1.01) | 3.81(1.13) |
| **Excited** | **2.90(1.64)** | **3.15(1.74)** | **4.10(1.55)** | **4.06(1.50)** | **2.93(1.13)** | **3.05(1.07)** | **2.53(1.13)** | **2.64(1.27)** |
| Focused | 2.96(1.59) | 4.03(1.57) | 3.75(1.63) | 4.29(1.41) | 2.30(0.87) | 3.62(1.17) | 2.04(0.92) | 2.89(1.40) |
| Glad | 3.41(1.57) | 4.26(1.41) | 4.10(1.43) | 4.40(1.33) | 3.25(0.97) | 4.00(1.11) | 3.09(1.17) | 3.59(1.41) |
| **Happy** | **3.97(1.44)** | **4.33(1.37)** | **4.68(1.32)** | **4.64(1.25)** | **4.09(1.00)** | **4.01(1.01)** | **3.68(1.08)** | **3.83(1.16)** |
| Proud | 2.59(1.70) | 4.21(1.53) | 3.59(1.78) | 4.63(1.33) | 1.83(0.67) | 4.33(1.14) | 1.67(0.90) | 3.77(1.42) |
| **Refreshed** | **2.97(1.63)** | **4.15(1.47)** | **3.81(1.63)** | **4.54(1.29)** | **2.27(1.01)** | **4.39(1.04)** | **2.20(0.99)** | **3.67(1.30)** |
| **Relaxed** | **3.58(1.55)** | **3.93(1.54)** | **4.18(1.37)** | **4.30(1.42)** | **3.23(1.09)** | **3.39(1.05)** | **2.82(1.11)** | **2.99(1.24)** |
| **Relieved** | **2.97(1.63)** | **3.75(1.65)** | **3.79(1.60)** | **4.21(1.46)** | **2.59(1.08)** | **3.55(1.28)** | **2.11(1.10)** | **2.72(1.31)** |
| Soothed | 3.52(1.55) | 3.50(1.60) | 3.98(1.40) | 3.87(1.44) | 2.90(1.08) | 3.15(1.04) | 2.76(1.09) | 2.81(1.21) |
| **Negative** |  |  |  |  |  |  |  |  |
| Afraid | 2.13(1.53) | 1.89(1.41) | 2.92(1.73) | 2.73(1.74) | 1.75(0.89) | 1.44(0.56) | 1.39(0.63) | 1.28(0.49) |
| **Anxious** | **2.55(1.66)** | **2.13(1.60)** | **3.37(1.65)** | **3.10(1.73)** | **2.41(1.19)** | **1.73(0.76)** | **2.10(1.14)** | **1.53(0.83)** |
| Ashamed | 3.02(1.74) | 1.89(1.58) | 3.49(1.67) | 2.69(1.88) | 3.03(1.30) | 1.22(0.52) | 2.45(1.15) | 1.15(0.45) |
| **Bored** | **2.76(1.62)** | **2.50(1.60)** | **3.29(1.65)** | **3.12(1.61)** | **3.06(1.33)** | **2.55(1.03)** | **2.36(1.17)** | **2.00(0.98)** |
| **Depressed** | **2.70(1.67)** | **2.17(1.58)** | **3.21(1.69)** | **2.88(1.72)** | **2.36(1.26)** | **1.61(0.73)** | **1.98(0.96)** | **1.48(0.65)** |
| Deprived | 2.32(1.61) | 2.35(1.56) | 3.31(1.72) | 3.25(1.69) | 1.97(0.93) | 1.91(0.98) | 1.64(0.69) | 1.62(0.83) |
| Disgusting | 2.62(1.71) | 1.92(1.51) | 3.34(1.66) | 2.83(1.85) | 2.78(1.23) | 1.53(0.81) | 2.07(1.14) | 1.16(0.46) |
| **Down** | **2.70(1.65)** | **2.01(1.44)** | **3.32(1.57)** | **2.97(1.69)** | **2.49(1.04)** | **1.87(0.75)** | **2.16(0.94)** | **1.63(0.85)** |
| **Frustrated** | **2.64(1.68)** | **2.17(1.64)** | **3.25(1.73)** | **3.05(1.73)** | **2.41(1.25)** | **1.74(0.90)** | **2.12(1.18)** | **1.50(0.73)** |
| Irritable | 2.40(1.51) | 2.17(1.56) | 3.10(1.67) | 2.91(1.69) | 2.28(1.09) | 1.84(0.79) | 2.14(1.05) | 1.69(0.89) |
| Lazy | 3.02(1.70) | 2.08(1.60) | 3.74(1.60) | 2.87(1.70) | 3.72(1.26) | 1.76(0.80) | 2.64(1.26) | 1.48(0.76) |
| **Numb** | **2.60(1.73)** | **2.09(1.62)** | **3.36(1.71)** | **3.09(1.80)** | **1.85(1.00)** | **1.48(0.63)** | **1.67(0.95)** | **1.43(0.76)** |
| Regretful | 3.20(1.73) | 2.04(1.60) | 3.66(1.63) | 2.93(1.81) | 3.29(1.42) | 1.36(0.57) | 2.63(1.26) | 1.35(0.66) |
| Sluggish | 3.16(1.61) | 2.07(1.53) | 3.87(1.53) | 3.08(1.75) | 3.44(1.28) | 1.64(0.63) | 2.97(1.22) | 1.51(0.66) |
| **Tired** | **2.80(1.57)** | **2.13(1.54)** | **3.48(1.56)** | **2.94(1.62)** | **3.21(1.04)** | **2.09(0.83)** | **2.83(1.21)** | **1.88(0.92)** |
| Worried | 2.53(1.61) | 2.00(1.50) | 3.16(1.68) | 2.77(1.71) | 2.48(1.17) | 1.71(0.80) | 2.07(0.99) | 1.58(0.79) |

*Notes:* Items selected for the brief form are bolded. HPF = Highly-processed-food expectancies, MPF = Minimally-processed-food expectancies
