# Supplementary material for: Development and validation of a brief form of the Anticipated Effects of Food Scale
Source: Appetite. Author manuscript; Available in PMC 2026 Feb 1. (PMC12314748; doi:10.1016/j.appet.2024.107843)
Supplement: Supplementary Tables 2 & 3 [file NIHMS2098013-supplement-Supplementary_Tables_2___3.docx]

Table S2

*Correlation coefficients of Anticipated Effects of Food Scale items with eating behaviours from Project 1*

|  | Intake of  added sugars | | Symptoms of  food addiction | | Emotional  eating | | Eating to cope  motives | |
| --- | --- | --- | --- | --- | --- | --- | --- | --- |
|  | **HPF** | **MPF** | **HPF** | **MPF** | **HPF** | **MPF** | **HPF** | **MPF** |
| **Positive** |  |  |  |  |  |  |  |  |
| Alert | .05 | .00 | .43*** | .25*** | .36*** | .23*** | .39*** | .18*** |
| Calm | .06 | -.05 | .33*** | .16* | .29*** | .10 | .34*** | .10 |
| **Cheerful** | **.11** | **-.05** | **.33***** | **.20**** | **.34***** | **.20**** | **.37***** | **.15*** |
| **Comforted** | **.15*** | **.04** | **.33***** | **.23***** | **.43***** | **.14*** | **.47***** | **.14*** |
| Content | .12 | -.11 | .22*** | .07 | .22*** | .03 | .26*** | .03 |
| Energized | .17** | -.08 | .47*** | .10 | .36*** | .09 | .43*** | .08 |
| **Excited** | **.22***** | **.05** | **.53***** | **.38***** | **.47***** | **.27***** | **.50***** | **.34***** |
| Focused | .15* | -.10 | .50*** | .18** | .38*** | .13* | .44*** | .10 |
| Glad | .08 | -.03 | .41*** | .15* | .40*** | .13* | .47*** | .13* |
| **Happy** | **.15*** | **-.01** | **.25***** | **.10** | **.38***** | **.08** | **.37***** | **.04** |
| Proud | .19** | -.08 | .64*** | .19** | .45*** | .23*** | .51*** | .17* |
| **Refreshed** | **.17**** | **-.12** | **.54***** | **.20**** | **.44***** | **.12** | **.48***** | **.11** |
| **Relaxed** | **.02** | **.00** | **.35***** | **.15*** | **.36***** | **.11** | **.40***** | **.10** |
| **Relieved** | **.15*** | **.00** | **.60***** | **.32***** | **.54***** | **.33***** | **.62***** | **.28***** |
| Soothed | .06 | .07 | .34*** | .24*** | .47*** | .19** | .40*** | .16* |
| **Negative** |  |  |  |  |  |  |  |  |
| Afraid | .19** | .22*** | .65*** | .73*** | .47*** | .51*** | .46*** | .57*** |
| **Anxious** | **.12** | **.23***** | **.59***** | **.76***** | **.53***** | **.58***** | **.50***** | **.64***** |
| Ashamed | .04 | .23*** | .48*** | .74*** | .49*** | .50*** | .41*** | .52*** |
| **Bored** | **.25***** | **.25***** | **.53***** | **.62***** | **.44***** | **.59***** | **.46***** | **.58***** |
| **Depressed** | **.16*** | **.29***** | **.50***** | **.70***** | **.51***** | **.59***** | **.48***** | **.64***** |
| Deprived | .15* | .22*** | .62*** | .64*** | .50*** | .58*** | .49*** | .57*** |
| Disgusting | .05 | .27*** | .50*** | .75*** | .46*** | .54*** | .40*** | .59*** |
| **Down** | **.11** | **.21***** | **.52***** | **.72***** | **.52***** | **.56***** | **.42***** | **.60***** |
| **Frustrated** | **.19**** | **.22***** | **.51***** | **.76***** | **.47***** | **.57***** | **.41***** | **.62***** |
| Irritable | .22*** | .17*** | .45*** | .68*** | .44**** | .55*** | .40*** | .58*** |
| Lazy | .21*** | .25*** | .29*** | .71*** | .34*** | .52*** | .31*** | .56*** |
| **Numb** | **.19**** | **.23***** | **.66***** | **.75***** | **.63***** | **.56***** | **.59***** | **.61***** |
| Regretful | .01 | .21*** | .36*** | .76*** | .41*** | .55*** | .35*** | .60*** |
| Sluggish | .12 | .30*** | .38*** | .75*** | .41*** | .53*** | .31*** | .58*** |
| **Tired** | **.23***** | **.26***** | **.43***** | **.72***** | **.46***** | **.57***** | **.44***** | **.60***** |
| Worried | .14* | .22*** | .53*** | .75*** | .51*** | .57*** | .46*** | .61*** |

*Notes:* Items selected for the brief form are bolded. HPF = Highly-processed-food expectancies, MPF = Minimally-processed-food expectancies, **p* < .05, ***p* < .01, ****p* <.001

Table S3

*Correlation coefficients of Anticipated Effects of Food Scale items with eating behaviours from Project 2*

|  | Symptoms of  food addiction | |
| --- | --- | --- |
|  | **HPF** | **MPF** |
| **Positive** |  |  |
| Alert | .53*** | .36*** |
| Calm | .39*** | .24*** |
| **Cheerful** | **.40***** | **.28***** |
| **Comforted** | **.25***** | **.30***** |
| Content | .18*** | .16*** |
| Energized | .54*** | .16*** |
| **Excited** | **.46***** | **.42***** |
| Focused | .58*** | .34*** |
| Glad | .37*** | .18*** |
| **Happy** | **.27***** | **.24***** |
| Proud | .61*** | .08 |
| **Refreshed** | **.51***** | **.21***** |
| **Relaxed** | **.40***** | **.32***** |
| **Relieved** | **.48***** | **.29***** |
| Soothed | .38*** | .35*** |
| **Negative** |  |  |
| Afraid | .63*** | .67*** |
| **Anxious** | **.55***** | **.60***** |
| Ashamed | .37*** | .68*** |
| **Bored** | **.40***** | **.49***** |
| **Depressed** | **.47***** | **.62***** |
| Deprived | .62*** | .55*** |
| Disgusting | .45*** | .62*** |
| **Down** | **.49***** | **.66***** |
| **Frustrated** | **.53***** | **.62***** |
| Irritable | .55*** | .59*** |
| Lazy | .24*** | .64*** |
| **Numb** | **.64***** | **.70***** |
| Regretful | .40*** | .66*** |
| Sluggish | .30*** | .67*** |
| **Tired** | **.38***** | **.59***** |
| Worried | .51*** | .62*** |

*Notes:* Items selected for the brief form are bolded. HPF = Highly-processed-food expectancies, MPF = Minimally-processed-food expectancies, **p* < .05, ***p* < .01, ****p* <.001
